# Application of ECIS to Evaluate the Effects of Porcine Urinary Bladder Matrix Hydrogels on Caco-2 Cell Attachment, Migration, and Barrier Formation

**DOI:** 10.3390/gels12060552

**Published:** 2026-06-19

**Authors:** Wei-Ling Chen, Chi-Tien Chen, Huynh-Quang-Dieu Nguyen, Phenpitcha Charoensaensuk, Chen-Yu Kao, Chun-Min Lo

**Affiliations:** 1 Department of Biomedical Engineering, National Yang Ming Chiao Tung University, Taipei 11221, Taiwan; bet1233.be11@nycu.edu.tw (W.-L.C.); chitien0407@gmail.com (C.-T.C.); 2 Graduate Institute of Applied Science and Technology, National Taiwan University of Science and Technology, Taipei 10607, Taiwan; d10822805@mail.ntust.edu.tw; 3 Graduate Institute of Biomedical Engineering, National Taiwan University of Science and Technology, Taipei 10607, Taiwan; phenpitchacream@outlook.com; 4 Department of Biomedical Engineering, National Defense Medical University, Taipei 11490, Taiwan

**Keywords:** urinary bladder matrix, decellularization, hydrogel, collagen, Caco-2, ECIS, electric fence, TEER

## Abstract

Recent studies have highlighted the potential of urinary bladder matrix (UBM) derived from decellularized porcine urinary bladder as a bioactive hydrogel. Despite its complex composition of over 100 proteins, Type I collagen is the primary constituent of UBM. Caco-2 cells are widely used as an in vitro model of the intestinal epithelium; however, to date, no published study has evaluated the effects of UBM on Caco-2 cells. In this study, Electric Cell–Substrate Impedance Sensing (ECIS) was used to measure Caco-2 cell attachment and wound-healing migration on UBM-coated microelectrodes. Our results demonstrate that UBM hydrogel coating at 0.2 mg/mL significantly accelerates cell attachment and enhances migration rates compared to uncoated controls. These stimulatory effects were comparable to those observed with 0.2 mg/mL Type I collagen, suggesting that UBM can function as effectively as Type I collagen. We further monitored barrier formation in Caco-2 cells cultured on UBM-coated transwell membrane inserts using TEER measurements and scanning electron microscopy. The TEER values reached 300 Ω·cm^2^ within three days, indicating the rapid establishment of mature tight junctions. Overall, these results show that UBM hydrogel coatings are effective substrates for Caco-2 cells, performing as well as Type I collagen in all our tests.

## 1. Introduction

Extracellular Matrix (ECM) is a complex three-dimensional network composed of collagen, glycosaminoglycans (GAGs), fibronectin, and various functional proteins [1,2]. ECM also plays a critical role in tissues and organs, providing not only physical structural support but also acting as a central regulator in orchestrating cellular behaviors and mediating signal transduction [3]. In recent years, decellularized ECM (dECM) has emerged as a focal point in tissue engineering [4]. Through chemical, physical, or enzymatic treatments, dECM is produced by removing cellular components and immunogenic substances while preserving the native ECM’s 3D architecture and bioactive constituents [5,6,7]. Compared with synthetic materials, dECM exhibits superior biocompatibility and low immunogenicity, providing cells with a physiological environment that closely mimics in vivo conditions. Consequently, it has been widely applied in tissue regeneration and repair, as well as in the establishment of in vitro pathological models [8,9].

Because of their stable supply and ease of procurement, porcine tissues have become the primary source for dECM preparation. Among the ECM products acquired from different porcine tissues after decellularization, the urinary bladder matrix (UBM) is widely used in vascular regeneration and various regenerative medicine studies owing to its excellent biocompatibility and its inductive properties for tissue remodeling [10,11,12]. Decellularized porcine UBM can effectively promote wound healing and tissue regeneration through its immunomodulatory functions [13,14,15]. In our previous studies, we successfully used sodium dodecyl sulfate (SDS) for decellularization. We formulated the resulting matrix into a dECM hydrogel and conducted a comprehensive analysis of its physical properties and biocompatibility [16]. These characteristics underscore the effectiveness of SDS-based decellularization in generating functional ECM hydrogels suitable for regenerative medicine. Furthermore, our studies have confirmed that UBM possesses the biological potential to promote the myogenic differentiation of C2C12 cells [17]. Variations in tissue selection during decellularization may significantly influence the resulting hydrogel’s composition, mechanical properties, and biological performance. Understanding how different regions or layers of the bladder contribute to these characteristics is essential for optimizing ECM hydrogel design and ensuring consistent functionality in biomedical applications [17].

Caco-2 cells, an immortal cell line derived from a human colorectal adenocarcinoma, are widely used as a cell model to study the intestinal epithelial barrier and drug permeability [18,19]. Type I collagen-coated surfaces have been shown to enhance Caco-2 adhesion, growth, and barrier formation [20,21]. Since the main component of UBM hydrogels is type I collagen, one may predict that Caco-2 cells cultured on UBM behave similarly to Caco-2 cells on type I collagen. However, the interaction between UBM and intestinal epithelial cells remains poorly understood. In particular, no systematic evaluation has been conducted to determine how UBM influences key epithelial functions such as cell attachment, migratory capacity, and barrier formation in Caco-2 cells. This represents a critical knowledge gap, as epithelial–matrix interactions are known to play a central role in maintaining intestinal homeostasis and facilitating mucosal repair following injury. The central hypothesis in this work is that UBM provides biologically relevant cues that enhance Caco-2 cell attachment, promote coordinated cell migration, and improve barrier formation compared to conventional culture substrates. This hypothesis is supported by evidence from other tissue systems in which ECM-derived scaffolds enhance integrin-mediated adhesion, cytoskeletal organization, and tight junction assembly [22]. Translating these principles to intestinal epithelial models could significantly improve the physiological relevance of in vitro barrier systems.

The interactions between cells and their substrates, including attachment, migration, and differentiation, involve a complex sequence of events that occur in an integrated, coordinated manner. However, conventional endpoint assays often fail to capture the continuous, highly dynamic transitions in cellular responses. Thus, real-time monitoring technologies are needed for investigating these cell–substrate interactions. Electric cell–substrate impedance sensing (ECIS) was developed by Giaever and Keese in 1984 and can be used to study the characteristics of anchorage-dependent cultured cell lines [23]. By culturing cells on micro-gold film electrodes and monitoring impedance changes caused by adherent cells, one can quantify changes in cell morphology and motility with exquisite sensitivity and in a noninvasive manner [24,25,26,27]. The ECIS method enables label-free quantification of morphological parameters, including membrane capacitance (Cm), the parameter α which relates to cleft resistance, and the junctional resistance between cells (Rb) [24,28].

For the quantitative assessment of cell migration, the ECIS electric fence (EF) technique can be utilized. During the initial stage of cell seeding, continuous high-current pulses are applied to the working electrode to inhibit cell attachment and spreading on its surface. This treatment forces the cells to grow and form a confluent monolayer in the area surrounding the working electrode. Once the pulses are deactivated, the surrounding cells begin to migrate into the vacant electrode area. As electrode coverage increases, impedance rises, enabling evaluation of the cell migration rate [29]. The effect of cell–matrix interactions on Caco-2 cell differentiation, particularly on barrier formation, can be assessed using transepithelial electrical resistance (TEER) [30,31]. The ECIS-TEER system, combined with standard 24-well transwell membrane inserts, enables continuous impedance measurements of barrier formation during incubation [32]. This integrated system uses specialized transfilter arrays with an embedded gold-film electrode at the bottom of each well. Using culture medium as the electrolyte, the weak AC passes through the cell monolayer cultured on the insert membrane between the top-dipping pin electrode and the bottom counter electrode.

In this study, Caco-2 cells, which exhibit robust barrier properties, were selected as the model system. The cells were cultured on surfaces coated with either collagen or UBM hydrogel coatings. We applied the ECIS system to evaluate the effects of UBM on the attachment and migration behaviors of Caco-2 cells. In addition, ECIS–TEER measurements enabled real-time monitoring of Caco-2 monolayer formation and quantitative assessment of barrier function. Finally, the results obtained from the UBM group were compared with those of the standard collagen group to evaluate the potential advantages of UBM as a biomimetic substrate.

## 2. Results and Discussion

### 2.1. Effects of UBM on Caco-2 Cell Attachment and Spreading

**Figure 1:**
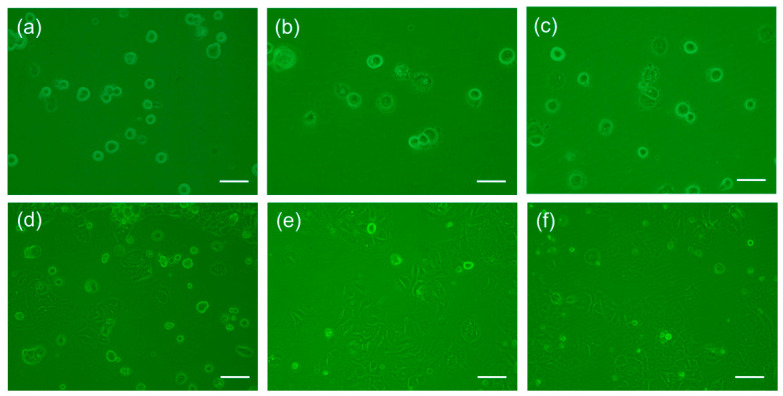
Phase contrast images of Caco-2 cell attachment 1 h (**a**–**c**) and 24 h (**d**–**f**) after seeding on culture wells with different ECM coatings: (**a**,**d**) uncoated control, (**b**,**e**) 0.2 mg/mL collagen, and (**c**,**f**) 0.2 mg/mL UBM. The scale bar is 100 μm in length.

Attachment to the extracellular matrix (ECM) is a fundamental cellular characteristic that governs critical processes, including migration, proliferation, and differentiation. To evaluate the bioactivity of our prepared matrices, we coated well plates with 0.2 mg/mL UBM and 0.2 mg/mL Type I collagen, respectively. Microscopic observation after 1 h and 24 h of incubation (Figure 1) revealed that Caco-2 cells adhered similarly and significantly faster on both ECM-coated surfaces than on uncoated controls.

Cellular areas were quantified using ImageJ 1.54k (NIH, Bethesda, MD, USA), and the equivalent radii (r) were derived from the measured areas (A) using the equation r = (A/π)^1/2^. Here, the cell shape was assumed to be rounded, and only individual cells without overlap were counted. Specifically, the average radii of Caco-2 cells 1 h after seeding on UBM-coated, collagen-coated, and uncoated culture wells were 6.1 ± 0.2 μm (*n* = 5), 5.8 ± 0.3 μm (*n* = 7), and 4.8 ± 0.2 μm (*n* = 5), respectively. Furthermore, as shown in Figure 1d–f, cells on the ECM coatings 24 h after seeding exhibited more extensive spreading with regular peripheries and a more compact cellular arrangement, suggesting superior biocompatibility and cell–substrate affinity. It has been reported that the rates of both spreading and proliferation of Caco-2 cells seeded onto type I collagen were substantially higher than those on culture plastic [33]. Since the major protein in porcine UBM is type I collagen [17], it is reasonable that UBM promotes Caco-2 cell attachment and may therefore provide a supportive microenvironment for intestinal epithelial cells.

**Figure 2:**
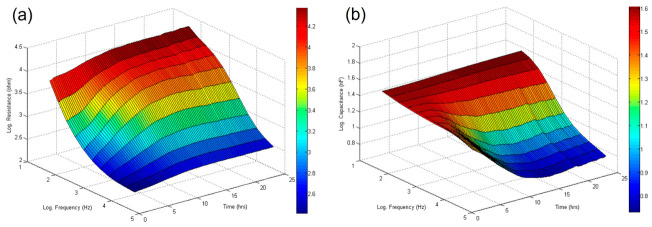
Typical three-dimensional plots obtained from MFT measurements of Caco-2 cell attachment and spreading, representing the changes in (**a**) resistance and (**b**) capacitance as a function of time and frequency. Here, cells were inoculated into 8W10E wells without coating at time zero, and the seeding density is 8.5 × 10^4^/cm^2^.

To examine the dynamics of Caco-2 cell attachment and spreading on UBM, we coated 8W10E ECIS array electrodes with UBM at various concentrations and with 0.2 mg/mL type I collagen. The 8W10E contains ten 250-μm diameter electrodes connected in parallel, which is good for monitoring a confluent cell layer. By averaging the signal across 10 electrodes, this array provides broader sample coverage and a more homogeneous impedance measurement. We inoculated cells into electrode wells at an 8.5 × 10^4^ cells/cm^2^ cell density and monitored the electrical impedances of the cell-covered electrodes for 24 h using multiple frequency time-Series (MFT) measurement. As shown in Figure 2, changes in measured resistance and capacitance as a function of frequency and time were obtained. Similarly to MDCK cell attachment and spreading data, the optimal detection frequencies for tracing resistance and capacitance changes in Caco-2 cells are 500 Hz and 64 kHz, respectively [28].

In general, MFT measurements using 8W10E or 8W1E electrode wells display a similar profile in the three-dimensional impedance spectrum. However, the measured resistance at 500 Hz using an 8W10E well is about 10 times lower than that using an 8W1E well, and the measured capacitance at 64 kHz is about 10 times higher. These differences are due to the total sensing electrode area of an 8W10E well being ten times that of an 8W1E well.

**Figure 3:**
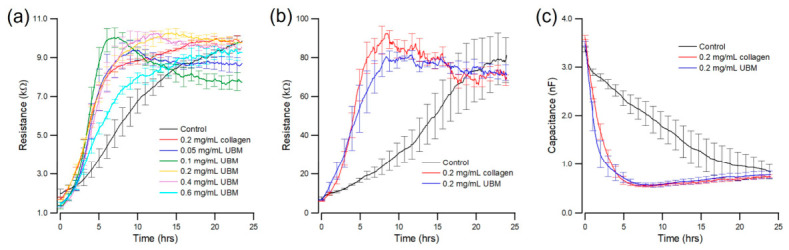
Resistance time series measured at 500 Hz showing the attachment and spreading of Caco-2 cells on different coated substrates using (**a**) 8W10E arrays (*n* = 4 for each condition) and (**b**) 8W1E arrays (*n* = 8 for each condition). (**c**) Capacitance time series measured at 64 kHz using 8W1E arrays to show Caco-2 cell–substrate coverage on different coated substrates (*n* = 8 for each condition). Uncoated electrodes were used as a control. Data were presented as mean ± standard error.

Figure 3a shows typical time series tracings at 500 Hz obtained from Caco-2 cells cultured on 8W10E array wells precoated with various concentrations of UBM. As shown in Figure 3a, responses varied with the ECM coating on the ECIS electrodes. The initial rapid rise in each curve was due to suspended Caco-2 cells settling, attaching, and spreading to form a monolayer, effectively blocking the current flow. After cell inoculation, the resistances of the 0.2 mg/mL collagen-coated and 0.05–0.4 mg/mL UBM-coated electrodes increased more rapidly over time than those of the 0.6 mg/mL UBM-coated electrodes and the uncoated control. We also repeated the same cell attachment and spreading experiment using an 8W1E array to increase measurement sensitivity. Here, we only compare 0.2 mg/mL collagen coating, 0.2 mg/mL UBM coating, and a control without coating.

The reason that 0.2 mg/mL collagen I was selected is that it falls within the range commonly used for collagen-coated intestinal epithelial cultures and provides robust, reproducible attachment and monolayer formation on porous transwell membranes [34]. Porcine UBM is primarily composed of collagen I, but also contains other basement membrane proteins, GAGs, and growth factors [35]. Comparing UBM with purified Type I collagen enables assessment of whether the biological complexity of a native ECM scaffold yields cellular responses comparable to those achieved with a collagen-only matrix.

For the control, the adsorbed proteins on electrodes were those present in the serum used to supplement the culture medium. Figure 3b,c show typical 24 h time series of resistance and capacitance measured at 500 Hz and 64 kHz. As shown in Figure 3b, by ~10 h, the measured resistances of collagen-coated and UBM-coated electrodes almost simultaneously reached a peak of ~80 kΩ, indicating the formation of a barrier function similar to that of MDCK cells [36]. At the same time, the resistance of the uncoated electrode only reached ~30 kΩ, much slower with time than that of the other two electrodes. The measured capacitances of collagen-coated and UBM-coated electrodes rapidly reached a minimum value of ~0.7 nF at 5 h, indicating the formation of a confluent Caco-2 cell layer (Figure 3c) [36]. In contrast, capacitance decreased slowly for the uncoated electrode. These results are consistent with microscopic observations as shown in Figure 1.

Rates of mammalian cell attachment and spreading are well known to depend on the type of protein coating on the substratum [22]. Indeed, studies using type I collagen coating have shown rapid, reliable cell adhesion in epithelial cells such as Caco-2 and MDCK cells [20,37]. This concept is also clearly reflected in the ECIS measurement as shown in Figure 3. Together, these results demonstrate that UBM and type I collagen coatings significantly accelerate the initial attachment and spreading of Caco-2 cells compared to the control. We showed that UBM performs as well as type I collagen for the attachment and spreading of Caco-2 cells.

### 2.2. Confirmation of Protein Adsorption Using BCA Assay

**Figure 4:**
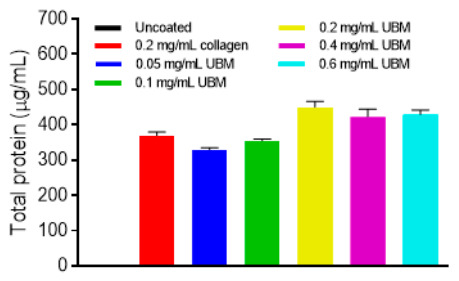
Quantification of the absorbed total protein on the ECM-coated well surface using the BCA protein assay. The uncoated well surface shows no protein absorption and serves as the negative control for comparison. Three independent experiments were performed (*n* = 3), and the results were presented as mean ± standard error.

A BCA (bicinchoninic acid) protein assay was used to determine the amount of protein that binds to the 12-well plate surface at various UBM coating concentrations. As shown in Figure 4, the absorbed total protein significantly increased in a nonlinear manner from 0.05 to 0.1 to 0.2 mg/mL in UBM coatings (*p* < 0.05). However, there was no significant difference in absorbed total protein between 0.2, 0.4, and 0.6 mg/mL of UBM coatings, indicating that surface binding saturation was probably reached. Thus, 0.2 mg/mL was selected as the optimal coating concentration in this study. Previously, we showed that UBM is a complex ECM composed of collagen, glycoproteins, and GAGs. In addition, UBM is not a uniform surface, but a heterogeneous protein network [16,17]. UBM adsorption might depend on the availability of binding domains and their structure, not just the amount of coating. This behavior directly explains why the protein absorption vs. UBM concentration curve is nonlinear and often saturates or even decreases at high coating concentrations.

### 2.3. Effects of UBM on the Morphological Parameters of Caco-2 Cells

**Figure 5:**
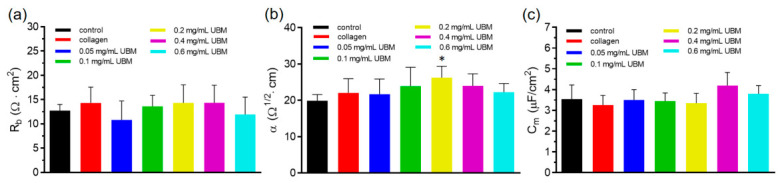
Morphological parameters of Caco-2 monolayers across different coated substrates obtained from frequency scan measurements and cell-electrode model calculation. Uncoated electrodes were used as a control. These three parameters are (**a**) junctional resistance between cells (Rb), (**b**) parameter α, and (**c**) membrane capacitance (Cm). Values were presented as mean ± standard error (*n* = 3). * *p* < 0.05.

The morphological properties of Caco-2 monolayers 24 h after seeding on 0.2 mg/mL of collagen I and UBM at various concentrations were further quantified using frequency-scan measurements and cell-electrode modeling [24,28]. After fitting the calculated values to the measured impedance spectrum, the junctional resistance between cells (Rb), the parameter α, and membrane capacitance (Cm) were determined and shown in Figure 5. While Rb and Cm values in Caco-2 cells were comparable across all substrates tested, the 0.2 mg/mL UBM group showed the highest α value (~26 Ω^1/2^·cm) among the coatings. Such a high α value reflects strong cell attachment and the high cleft resistance (α^2^) between the basal membrane and substrate. In addition to the ~50% of collagen, porcine UBM hydrogels contain ~30% of keratin and a small amount of other ECM proteins [17]. This biochemical composition might explain the UBM hydrogel’s greater ability to support the attachment and spreading of Caco-2 cells.

The α value of Caco-2 cells is close to that of MDCK cell monolayers [28]. However, the Rb values of Caco-2 cells either cultured on 0.2 mg/ML collagen I or on 0.2 mg/mL UBM are close to 14 Ω·cm^2^, which is much smaller than that of MDCK cells (~55 Ω·cm^2^) [28]. Interestingly, varying the UBM concentration did not yield substantial changes in Rb parameters. This finding implies that Caco-2 cells form a functionally equivalent epithelial barrier on UBM hydrogel coatings as they do on 0.2 mg/mL of type I collagen matrices.

### 2.4. Effects of UBM on Caco-2 Cell Migration

To evaluate the effects of 0.2 mg/mL collagen I and UBM coatings on Caco-2 cell migration, an ECIS electric fence (EF) was performed to create a localized cell-free zone on the electrodes. Unlike traditional wound-healing assays, high-frequency and high-current pulses were applied to the working electrodes to inhibit initial cell attachment specifically within the electrode area, without killing or scaping cells [29]. After cells attach everywhere else and form a confluent monolayer, the high-current pulse is turned off, and the attached neighboring cells start migrating into the previously protected cell-free zone. Cell migration was quantified using the T50 value, defined as the time required for cells to reach 50% of the maximum resistance plateau. Resistance time series were monitored at 500 Hz using 8W10E arrays.

**Figure 6:**
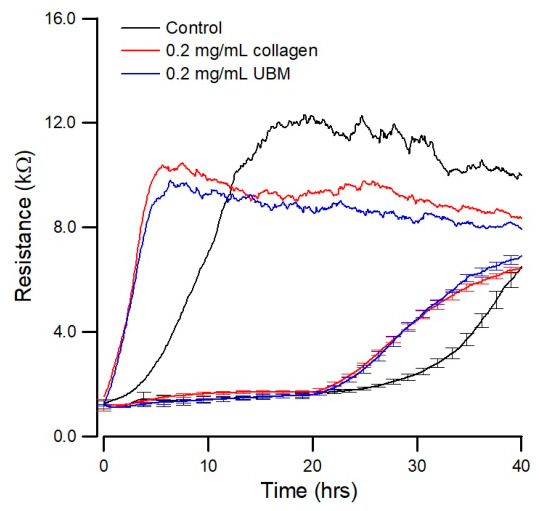
Analysis of Caco-2 cell migration on different substrates using the ECIS electric fence method and 8W10E arrays. The electric fence function prevents cells from attaching to the ECIS electrode for 20 h. After the electric fence is off, the cells migrate into the cell-free electrodes, and the cell migration kinetics are measured afterward. The resistance time series shown in the figure is measured at 500 Hz. Values in the recovery curves were presented as mean ± standard error (*n* = 3).

As shown in Figure 6, the top three time-series curves were monitored to verify the formation of confluent cell layers 20 h after seeding. The lower three time-series curves were used for T50 assessment. The T50 values for both 0.2 mg/mL UBM and 0.2 mg/mL Type I collagen were approximately 5 h, whereas the uncoated control group required 14 h. Recently, porcine UBM was clinically used to heal severe, complex wounds in patients who were poor candidates for traditional reconstructive surgery, such as local or free flaps [13]. The possible mechanism is that UBM acts as a biological scaffold, providing ECM proteins that encourage cell growth, migration, and tissue regeneration. Previous studies using Caco-2 cells have demonstrated that substrate-bound extracellular matrix proteins differentially regulate cell attachment, spreading, morphology, and migration [20,38]. Our results also indicate that Caco-2 cells exhibit significantly higher migration and coverage rates on type I collagen- and UBM-coated substrates than on non-coated surfaces. The EF method is particularly valuable for assessing how different substrates influence cell migration because the surface coating is preserved and dead-cell debris is avoided.

### 2.5. Effects of UBM on Caco-2 Cell Barrier

**Figure 7:**
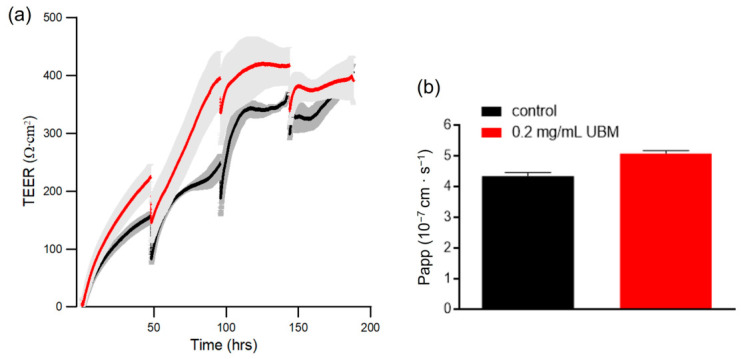
(**a**) Continuous measurement of TEER showing the accelerated barrier maturation of Caco-2 cells on UBM-coated transwell membranes. Resistance time series measured at 500 Hz showing TEER values for Caco-2 cells cultured on transwell membrane inserts with (red) or without (black) 0.2 mg/mL UBM coating. The culture medium was replaced every 2 days, and TEER data were collected by MFT measurement for 8 days. Curve values were presented as mean ± standard error (*n* = 3). The center line represents the average values, and the shaded error band shows the standard error across individual average value. (**b**) Phenol red permeability (Papp) measurement to confirm Caco-2 monolayer integrity 7 days post-seeding with or without UBM coating. Papp values were presented as mean ± standard error (*n* = 3).

As an in vitro model of the intestinal epithelium, Caco-2 cells can spontaneously differentiate into enterocyte-like monolayers with tight junctions. TEER measurement is a standard method for monitoring barrier integrity in the Caco-2 monolayer. To evaluate the efficacy of the UBM coating on Caco-2 cell culture in transwell inserts, we used ECIS-TEER to monitor cell growth and barrier formation. Caco-2 cells were seeded at a density of 8.5 × 10^4^ cells/cm^2^ onto insert membranes with or without 0.2 mg/mL UBM coating. A TEER value of ≥300 Ω·cm^2^ is a widely accepted, standard threshold for validating the integrity of Caco-2 cell monolayer [39]. As shown in Figure 7a, the UBM group exhibited an accelerated increase in TEER, reaching the threshold of 300 Ω·cm^2^ at approximately 75 h (day 3). In contrast, the control group reached the same resistance at 100 h (day 4). While both groups eventually established a functional monolayer with high integrity, the UBM coating significantly reduced the time required for barrier maturation, achieving the target resistance approximately 25 h earlier than the control.

**Figure 8:**
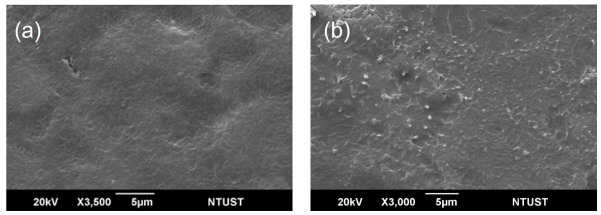
SEM images of the Caco-2 monolayer cultured on 0.2 mg/mL UBM-coated transwell membranes. (**a**) Microvilli were not found on day 14. (**b**) Standing microvilli were observed over several cell-covered areas on day 21 after seeding.

In addition to TEER measurement, we measured phenol red apparent permeability (Papp) to assess tight junction integrity in the Caco-2 cell monolayer (Figure 7b). Papp value of Caco-2 cells cultured for 7 days with or without UBM coating was approximately 5 × 10^−7^ cm/s (<1 × 10^−6^ cm/s), indicating that the Caco-2 monolayer is intact, and the tight junctions are closed. The differentiation morphology of Caco-2 cells cultured on UBM-coated transwell membrane inserts was also characterized using a scanning electron microscope. After culturing Caco-2 cells at a density of 8.5 × 10^4^ on UBM-coated transwell membranes for 7, 14, and 21 days, we also used SEM to examine the differentiated status of Caco-2 cells. Despite meeting the permeability requirements on day 7, microvilli were not observed on either day 7 or day 14 (Figure 8a), but were observed on day 21 (Figure 8b).

Type I collagen has been shown to provide a substrate that facilitates Caco-2 cell spreading and migration during early monolayer formation while simultaneously promoting integrin-dependent epithelial differentiation and establishment of a functional intestinal barrier [34,38,40]. The composition of the UBM used in this study (collagen, glycoproteins, GAGs) supports cell adhesion and motility signaling [16]. Our data demonstrate that UBM coating promotes both migration and barrier formation in Caco-2 cells. It is worth noting that, for epithelial cells such as Caco-2 cells, migration and barrier formation often occur sequentially. UBM coating enhances Caco-2 cell attachment and spreading (Figure 3), promotes migration to cover the available surface area (Figure 6), allowing cells to achieve confluence rapidly. Although migration-associated loosening of junctions occurs, this is typically temporary and followed by improved tight junction formation and stronger epithelial integrity over time [41].

While TEER measurement is useful for evaluating the barrier function of epithelial or endothelial cell layers in cell culture, careful interpretation is necessary. A high TEER value may partially result from reduced cell–substrate spacing, smaller pore size, lower pore density, or a lower AC frequency for sampling [42]. Several studies explicitly discussed the concept of cleft resistance (sometimes denoted R_cleft_ or represented by the ECIS parameter α^2^), which reflects the resistance of current flow in the narrow space between the basal cell membrane and the electrode/substrate [28,43,44]. One of our early works explicitly stated that TEER is not a pure readout of paracellular permeability, but rather a composite electrical measurement influenced by basal cell attachment and current pathways beneath the cells [42]. In this case, the TEER values might include cleft resistance due to cell–substrate contact (α^2^), which ECIS can determine [28]. For Caco-2 cells cultured on 0.2 mg/mL UBM, parameter α is ~26 Ω^1/2^·cm. Thus, cleft resistance (α^2^ can add additional resistance to TEER, as high as hundreds of Ω·cm^2^. Further development of cell-transwell-electrode models is needed to assess TEER values more accurately.

Decellularized porcine urinary bladder matrix (UBM) has emerged as a versatile extracellular matrix–based biomaterial with demonstrated utility in a wide range of regenerative medicine applications. Its clinical and preclinical success in wound healing, soft tissue reconstruction, hernia repair, and urologic tissue engineering has been attributed to its ability to provide a bioactive scaffold that supports constructive tissue remodeling, angiogenesis, and favorable host immune modulation [11,45]. Although direct studies of porcine UBM on Caco-2 intestinal epithelial cells are limited, extensive literature demonstrates that UBM-derived ECM scaffolds promote cell adhesion, migration, and constructive tissue remodeling across multiple cell types, including endothelial, fibroblast, and progenitor cell systems [46,47,48]. For example, previous studies using L929 cells in our laboratory and others have shown that UBM is generally non-cytotoxic, supports cell viability, promotes adhesion and migration, and stimulates fibroblast-mediated remodeling, making it suitable for wound-healing applications [16,48]. In addition, UBM enhances C2C12 proliferation and myogenic differentiation, indicating its potential as a bioactive scaffold for wound healing and skeletal muscle regeneration [16]. The consistent pro-regenerative effects of the UBM observed in diverse cell types suggest that its biological activity is not cell-type specific but rather reflects a broadly generalizable extracellular matrix-mediated mechanism.

This study provides preliminary insight into the influence of UBM on intestinal epithelial behavior. Our findings suggest that UBM supports Caco-2 cell attachment and promotes migratory activity, indicating a potentially favorable microenvironment for epithelial restitution. Furthermore, evidence of enhanced barrier formation implies that UBM may contribute to functional epithelial maturation, although the underlying mechanisms remain to be elucidated. Future work should include comparative studies with other extracellular matrix scaffolds and in vivo validation to establish physiological relevance. Additionally, investigating long-term barrier integrity, tight junction protein expression, and responses under inflammatory conditions would further clarify the translational potential of UBM in intestinal tissue engineering and regenerative medicine.

## 3. Conclusions

In this study, we applied ECIS to investigate the effects of porcine UBM hydrogel coatings on Caco-2 cell attachment, proliferation, migration, and barrier formation. As a label-free, real-time impedance-based cellular assay, ECIS demonstrates its versatility in monitoring changes in cell behavior. Our data, combined with microscopy observations and the apparent permeability coefficient of phenol red, indicate that the UBM coating may enhance these cellular functions in Caco-2 cells, as does 0.2 mg/mL type I collagen. Porcine urinary bladder matrix (UBM) hydrogels are promising biomaterials for regenerative medicine because they can serve as injectable, bioactive scaffolds that support cell growth, angiogenesis, and tissue repair.

## 4. Materials and Methods

### 4.1. The Preparation of UBM Hydrogel

The UBM hydrogel used in this study was derived from our laboratory stock and prepared as described in a previous study [16]. Briefly, UBM powder was digested in 0.01 N hydrochloric acid (HCl; Sigma Aldrich, St. Louis, MO, USA) containing 3 mg/mL pepsin for 48 h at room temperature to achieve a final UBM concentration of 10 mg/mL. The resulting enzymatic digest is hereafter referred to as “pre-gel.” For subsequent experiments, the pre-gel was further diluted in serum-free DMEM to achieve final UBM concentrations of 0.05, 0.1, 0.2, 0.4, and 0.6 mg/mL for surface coating. The effectiveness of the decellularization procedure was confirmed by DAPI staining, hematoxylin and eosin staining, dsDNA quantification, and DNA fragment size as described in our previous study [16].

### 4.2. Cell Culture

The human colon adenocarcinoma cell line C2BBe1 (a Caco-2 clone) was obtained from the Bioresource Collection and Research Center in Taiwan (BCRC Number 60182). The cells were cultured in Dulbecco’s Modified Eagle’s Medium with high glucose (DMEM-HG, 4.5 g/L; Gibco, Life Technologies, Rockville, MD, USA), supplemented with 10% fetal bovine serum (FBS; Gibco, Life Technologies, Rockville, MD, USA), 1% penicillin-streptomycin (Gibco, Life Technologies, Rockville, MD, USA), and 0.01 mg/mL human holotransferrin (Sigma Aldrich, St. Louis, MO, USA). The Caco-2 cells were cultured at 37 °C in a humidified atmosphere containing 5% CO_2_. Upon reaching 80% confluence, the cells were subcultured using a standard trypsin-EDTA procedure (Gibco, Life Technologies, Rockville, MD, USA).

### 4.3. Impedance Measurements Using ECIS

#### 4.3.1. Instrumentation

The ECIS Zθ instrument, associated software, and electrode arrays (8W1E and 8W10E) were purchased from Applied BioPhysics (Troy, NY, USA). Both array types consist of 8 wells, each with a growth area of 0.8 cm^2^. The 8W1E and 8W10E arrays, respectively, contain 1 and 10 sensing electrodes with 250-μm diameter per well. Before cell seeding, the arrays were plasma-treated for 90 s to clean the electrode surfaces and ensure sterility. The electrodes were coated with either various concentrations of UBM or 0.2 mg/mL Type I collagen (Corning 354236, Corning, NY, USA) for 1 h at 37 °C. To remove non-adherent proteins or peptides, each well was rinsed once with serum-free medium before cell inoculation.

#### 4.3.2. Multiple Frequency Time-Series (MFT) Measurement

Cell attachment and spreading were monitored using MFT measurement from initial cell seeding until the formation of a confluent cell layer. Each well was measured at 11 preset frequencies ranging from 62.5 Hz to 64 kHz [28]. Capacitance time series at 64 kHz and resistance time series at 500 Hz were used to illustrate the dynamic processes of Caco-2 cell attachment and spreading. If different AC frequencies are employed, the amount of current flowing through paracellular and transcellular pathways, and thus the measured impedance, will vary.

#### 4.3.3. Frequency Scan Measurement

To quantify morphological parameters of Caco-2 cells, the impedance of each electrode was measured at 25 frequencies ranging from 31.25 Hz to 100 kHz before and after cell monolayers were formed. Using a cell-electrode model, the impedance spectrum of a cell-covered electrode was calculated from the measured impedance spectrum of a cell-free electrode, with a few suitable morphological parameters [24,28]. These parameters include the junctional resistance between adjacent cells (Rb), the parameter α, and the cell membrane capacitance (Cm). After the measured impedance spectrum of the same electrode covered with cells was fitted with the calculated values, those morphological parameters can be determined.

#### 4.3.4. ECIS Electric Fence (EF) Assay

To study cell migration kinetics, an electric fence was set up immediately after cell inoculation. A series of high-current pulses was applied to the working electrodes of the 8W10E arrays to prevent cell attachment and spreading within the active electrode area. Specifically, three consecutive sinusoidal pulses (6 mA at 40 kHz, 200 ms duration) were delivered every 5 min. This electrical barrier was maintained until a confluent monolayer formed on the well’s surrounding non-electrode surface. Once confluence was achieved, the EF was deactivated, allowing cells to migrate into the previously cell-free electrode area. The subsequent recovery of impedance was recorded to quantify the migration kinetics. The speed can be calculated with the halfway recovery time from baseline to plateau (T50) [29].

#### 4.3.5. ECIS-TEER Measurement

Transwell polycarbonate membrane inserts (area: 0.33 cm^2^, pore size: 0.4 μm; Corning, Transwell^®^ #3413) were coated with UBM (0.2 mg/mL) and incubated for 1 h. The inserts were then rinsed once with culture medium to remove unbound ECM. Caco-2 cells were subsequently seeded at a density of 8.5 × 10^4^ cells/cm^2^ and connected to an ECIS^®^ 8-well Trans Filter Adapter (8W tFA). Cells were cultured for 8 days at 37 °C in a humidified atmosphere containing 5% CO_2_, and the culture medium was replaced every 2 days. During the culture period, transepithelial electrical resistance (TEER) was continuously monitored by MFT measurements to evaluate barrier formation. TEER values (shown as Ω·cm^2^) were calculated by subtracting blank filter resistance from the sample resistance and then multiplying the result by the area of effective membrane on the filter insert.

### 4.4. BCA Protein Assay

The total protein adsorption after surface coating was validated using the BCA (bicinchoninic acid) Protein Assay Kit (Bioman, New Taipei, Taiwan). The working reagent was prepared by combining Reagent A (BCA and buffer) and Reagent B (copper sulfate) at a 50:1 ratio. In a 12-well plate, 200 µL of UBM at different concentrations (0.05, 0.1, 0.2, 0.4, 0.6 mg/mL) and 0.2 mg/mL of collagen I were pipetted into individual wells and incubated for 1 h at 37 °C. Each coated sample was washed twice with sterile PBS, and 200 µL of working solution was added to each sample, which was further incubated for 30 min at 37 °C. After transferring 200 µL of the mixture into wells of a 96-well plate, the absorbance at 560 nm was measured using a plate reader. The total protein concentration of each coated sample was extrapolated against the standard curve created from a serial dilution of BSA (20–2000 μg/mL).

### 4.5. Assessment of Caco-2 Monolayer Integrity by Phenol Red Permeability

Caco-2 cells were cultured on Transwell^®^ #3413 inserts with or without UBM coating for 7 days. Monolayer integrity was evaluated by measuring the apparent permeability coefficient (Papp) of phenol red as a marker of paracellular transport. Following treatment, cell monolayers were washed three times with phosphate-buffered saline containing Ca^2+^ and Mg^2+^. Phenol red solution (1 mM, 0.5 mL) was added to the apical compartment, while 1.0 mL transport buffer was added to the basolateral compartment. After incubation for 60 min at 37 °C, samples were collected from the basolateral chamber and mixed with 1 N NaOH before absorbance was measured at 560 nm. The apparent permeability coefficient (Papp, cm/s) was calculated according to: Papp = (dQ/dt)/(A × C0), where dQ/dt is the rate of phenol red appearance in the receiver compartment, A is the membrane surface area, and C0 is the initial donor concentration. Monolayers exhibiting phenol red Papp values ≤ 1 × 10^−6^ cm/s were considered intact barrier integrity [39].

### 4.6. Scanning Electron Microscopy (SEM)

Cells cultured on UBM-coated transwell membrane inserts were rinsed with PBS and fixed in a modified Karnovsky fixative for 1 h. Samples were then dehydrated using a series of ethanol concentrations. Finally, samples were fully dehydrated using a critical-point dryer (PVT-3B, Tousimis, Rockville, MD, USA). The hydrated samples were sputter-coated with gold-palladium and examined by SEM (JSM-6390LV, JEOL, Tokyo, Japan).

### 4.7. Statistical Analysis

Statistical significance was evaluated using one-way or two-way analysis of variance (ANOVA) to determine the differences between the experimental and control groups (GraphPad Prism 8.0, GraphPad Software Inc., San Diego, CA, USA). All experiments were performed in at least triplicate, and data are presented as the mean± standard error of the mean (SEM). A *p*-value of less than 0.05 was considered statistically significant.

## Data Availability

The data presented in this study are available upon reasonable request from the corresponding authors.
